# Simulation on an Advanced Double-Sided Cooling Flip-Chip Packaging with Diamond Material for Gallium Oxide Devices

**DOI:** 10.3390/mi15010098

**Published:** 2024-01-03

**Authors:** He Guan, Dong Wang, Wentao Li, Duo Liu, Borui Deng, Xiang Qu

**Affiliations:** School of Microelectronics, Northwestern Polytechnical University, Xi’an 710129, China; wang.dong@mail.nwpu.edu.cn (D.W.); 1961175010@mail.nwpu.edu.cn (W.L.); liud2022@mail.nwpu.edu.cn (D.L.); dengborui@mail.nwpu.edu.cn (B.D.); quxiang2022@mail.nwpu.edu.cn (X.Q.)

**Keywords:** gallium oxide, flip-chip packaging, thermal simulation

## Abstract

Gallium oxide (Ga_2_O_3_) devices have shown remarkable potential for high-voltage, high-power, and low-loss power applications. However, thermal management of packaging for Ga_2_O_3_ devices becomes challenging due to the significant self-heating effect. In this paper, an advanced double-sided cooling flip-chip packaging structure for Ga_2_O_3_ devices was proposed and the overall packaging of Ga_2_O_3_ chips was researched by simulation in detail. The advanced double-sided cooling flip-chip packaging structure was formed by adding a layer of diamond material on top of the device based on the single-sided flip-chip structure. With a power density of 3.2 W/mm, it was observed that the maximum temperature of the Ga_2_O_3_ chip with the advanced double-sided cooling flip-chip packaging structure was 103 °C. Compared with traditional wire bonding packaging and single-sided cooling flip-chip packaging, the maximum temperature was reduced by about 12 °C and 7 °C, respectively. When the maximum temperature of the chip was controlled at 200 °C, the Ga_2_O_3_ chip with double-sided cooling packaging could reach a power density of 6.8 W/mm. Finally, by equipping the top of the package with additional water-cooling equipment, the maximum temperature was reduced to 186 °C. These findings highlight the effectiveness of the proposed flip-chip design with double-sided cooling in enhancing the heat dissipation capability of Ga_2_O_3_ chips, suggesting promising prospects for this advanced packaging structure.

## 1. Introduction

Third-generation semiconductor materials, with high breakdown voltage, electron mobility, thermal stability, and radiation resistance, are increasingly employed in the field of high-frequency, high-power, and high-integration electronic devices [1,2,3]. After years of development, power devices based on third-generation wide-band-gap semiconductor materials have gradually approached or even surpassed the performance of silicon-based semiconductor power devices [4,5]. Gallium oxide (Ga_2_O_3_), a new type of third-generation semiconductor material, has a larger band gap, higher breakdown field strength, and larger Baliga figure of merit. Compared with other commonly used third-generation wide-band-gap semiconductor materials such as GaN and SiC, Ga_2_O_3_ presents great potential for future applications in the fields of high-voltage, high-power, low-loss power devices due to its lower production cost [6,7,8,9].

However, the thermal conductivity of Ga_2_O_3_ is only 10–27 W/m K at room temperature. The low thermal conductivity prevents the heat inside the Ga_2_O_3_ devices from dissipating out rapidly, and the heat will accumulate, leading to the increase of the internal temperature of the device. The serious self-heating effect will limit the further improvement of the device power density and will even cause reliability problems of the device and reduce the operation life of the device [10,11,12]. Some studies have been reported to relieve the self-heating effect of Ga_2_O_3_ devices by optimizing the device structure and the process design. S. H. Kim et al. investigated the effects of anisotropic thermal conductivity of β-Ga_2_O_3_ and the geometric design of metal electrode interconnections on device self-heating [13], demonstrating the importance of device layout design of transverse β-Ga_2_O_3_ transistors to maximize the electrical and thermal properties. R. H. Montgomery et al. proposed the use of thermally conductive dielectrics on β-Ga_2_O_3_ MOSFETs with a vertical channel structure to increase the device power density [14]. B. Chatterjee et al. performed a thermal analysis of multi-fin Ga_2_O_3_ vertical transistors using infrared thermal microscopy and coupled electro-thermal modeling to investigate the self-heating behavior of Fin-FETs with different numbers of fins, fin design parameters, and device orientation to further reduce the thermal resistance [15].

In practical applications, in addition to researching the device itself to suppress the self-heating effect, more improvements were carried out in the thermal design of the chip package [16,17,18]. B. Chatterjee and K. Zeng et al. developed a three-dimensional coupled electro-thermal model based on the electro-thermal characterization results and tested the effectiveness of various active and passive cooling solutions [16]. C. Yuan et al. explored the limitations of various core-level thermal management schemes on Ga_2_O_3_ MOSFETs using numerical simulations and comprehensively investigated the effects of various cooling methods and material choices on the device channel temperature [17]. S. Kim et al. conducted a thermal modeling study on Ga_2_O_3_ vertical transistors and analyzed the effects of thermal management strategies on their thermal performances [18].

Based on the above-mentioned research, this paper further explored the thermal behaviors of Ga_2_O_3_ chip packaged by a Ga_2_O_3_ device, proposed an enhanced double-sided cooling flip-chip packaging structure based on diamond material, carried out the design of package structure and package thermal materials, and conducted a detailed study on the thermal performance of the overall package of this enhanced Ga_2_O_3_ chip through simulation. Compared with the traditional wire bonding packaging and single-sided cooling flip-chip packaging, it can be seen that the double-sided cooling flip-chip packaging structure proposed in this paper has a better effect on the heat dissipation of Ga_2_O_3_ chips and has a good application prospect.

## 2. Modeling of Wire Bonding and Single-Sided Cooling Flip-Chip Packaging

**Figure 1:**
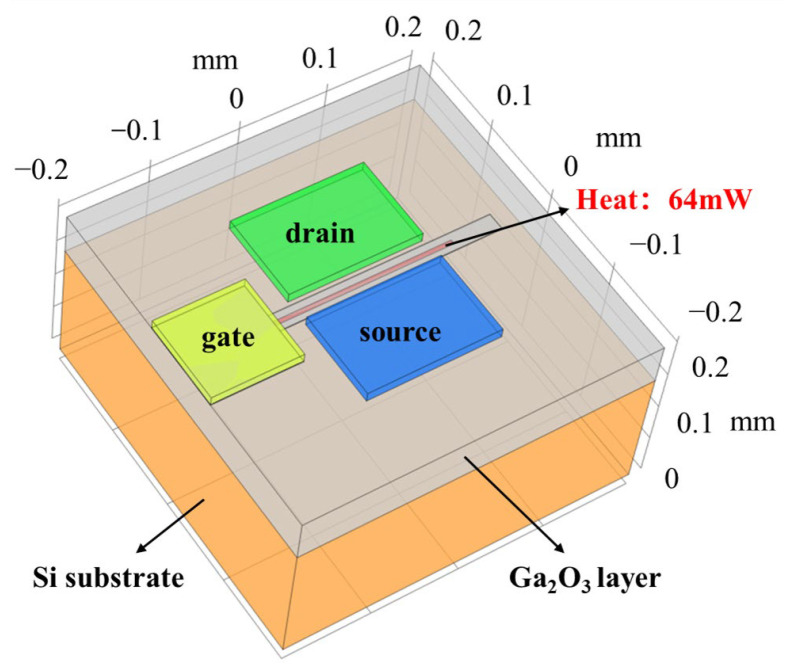
Ga_2_O_3_ device model.

In the design of the package, this paper refers to the Ga_2_O_3_ device, which had been reported in Ref. [3]. The bottom substrate of the device is Si of 0.15 mm, and the maximum drain current density (I_DS_) is 80 mA/mm at the V_DS_ of 40 V, which means the power density is 3.2 W/mm (L_DS_ = 20 μm). The device model structure is shown in Figure 1. In the simulation, the heat source was added at the channel of the active layer below the gate, the size of the heat source was set to 200 μm × 5 μm × 0.2 μm, and the heat dissipation rate was set to 64 mW.

**Figure 2:**
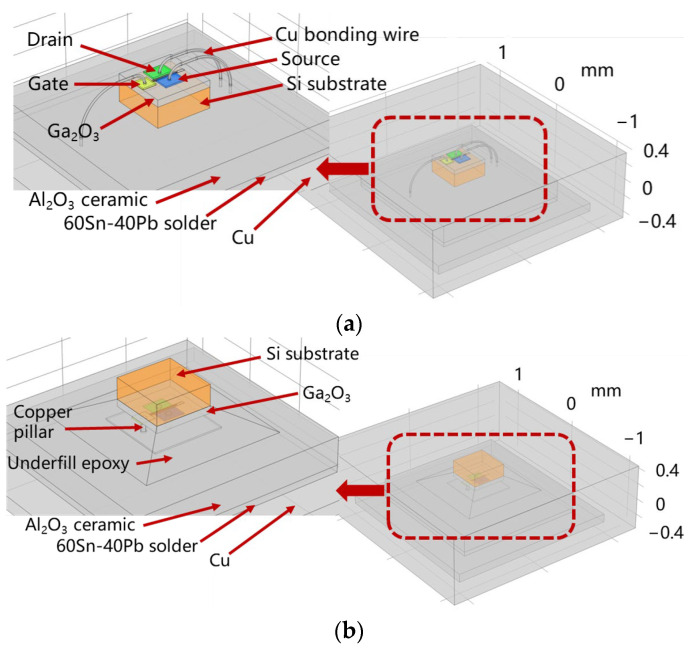
Simulated packaging model: (**a**) Wire bonding model; (**b**) single-sided cooling flip-chip model.

The structures of the wire bonding and single-sided cooling flip-chip models are shown in Figure 2a,b, respectively. The substrate of both the wire bonding and single-sided cooling flip-chip models was made of Al_2_O_3_ ceramic material, and a layer of Cu as an additional high thermal conductivity material was attached to the bottom of the substrate by solder. The outer package was made of epoxy molding plastic. Wire bonding was carried out with copper wire with a diameter of 20 μm. The device of the flip-chip packaging model was inverted on the substrate and electrically interconnected with the substrate through copper pillars with a radius of 20 μm and a height of 99 μm; then the device was fixed and protected with epoxy resin bottom-filling adhesive with 70% silica content. Detailed information on the size and material parameters of the package design is listed in Table 1.

**Figure 3:**
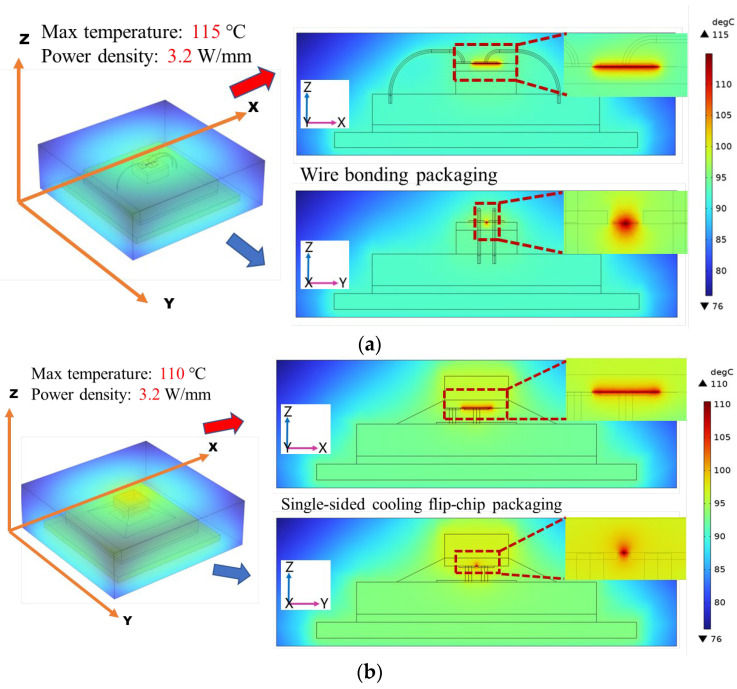
Thermal simulation results of wire bonding packaging and single-sided cooling flip-chip packaging. (**a**) Wire bonding packaging; (**b**) single-sided cooling flip-chip packaging.

The temperature distribution of the wire bonding packaging is shown in Figure 3a. The maximum temperature of the wire bonding packaging was 115 °C. The temperature distribution of the single-sided cooling flip-chip packaging is shown in Figure 3b. The maximum temperature of the flip-chip packaging was 110 °C, which was 5 °C lower than that of the wire bonding packaging. Therefore, the flip-chip packaging could improve the heat dissipation of the Ga_2_O_3_ chip effectively compared with the wire bonding packaging. This is because, in the flip-chip packaging, the heat generated by the chip can be directly transferred to the substrate through the interconnecting copper pillars, and then the heat is transferred to the bottom high thermal conductivity Cu layer.

## 3. Double-Sided Cooling Flip-Chip Packaging

**Figure 4:**
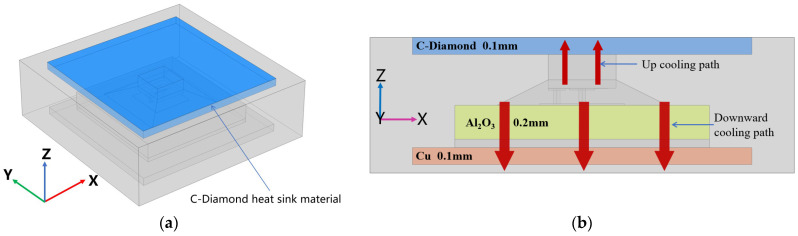
Schematic of the double-sided cooling flip-chip packaging model and heat dissipation path. (**a**) Double-sided cooling flip-chip packaging model; (**b**) schematic of the double-sided cooling heat dissipation path.

This paper proposes an advanced double-sided cooling flip-chip packaging structure for Ga_2_O_3_ devices. Based on the above single-sided flip-chip structure, a layer of high thermal conductivity material was added on top of the device to form a double-sided cooling heat dissipation structure, so that the heat of the device could be dissipated from the device down through the alumina ceramic and the Cu base plate on the bottom side and up through the Si substrate and high thermal conductivity material on the top side. The high thermal conductivity material above the device was diamond material with excellent thermal conductivity up to 2500 W/m·K, and the size of the diamond was 2 × 2 × 0.1 mm^3^. The advanced double-sided cooling flip-chip packaging model and the heat dissipation path are shown in Figure 4.

**Figure 5:**
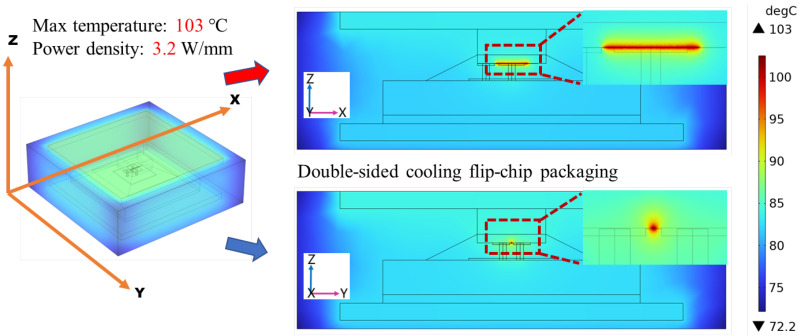
Thermal simulation result of enhanced double-sided cooling flip-chip Ga_2_O_3_ chip.

The thermal simulation result of the enhanced double-sided cooling flip-chip Ga_2_O_3_ chip is shown in Figure 5. The maximum temperature was at the active layer of the device, and it can be observed that heat is transferred both upwards and downwards simultaneously. The maximum temperature of the double-sided cooling flip-chip package was 103 °C; compared with the traditional wire bonding package in Figure 3a and the single-sided cooling flip-chip packaging in Figure 3b, the maximum temperature was reduced by about 12 °C and 7 °C, respectively. The simulation results of maximum temperature are shown in Table 2.

**Figure 6:**
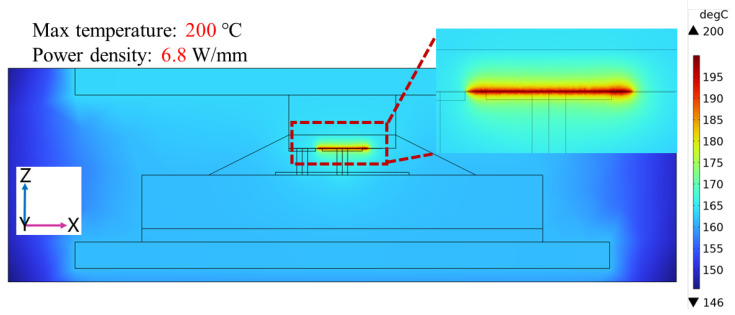
Simulation results of double-sided cooling flip-chip packaging model at a power density of 6.8 W/mm.

**Figure 7:**
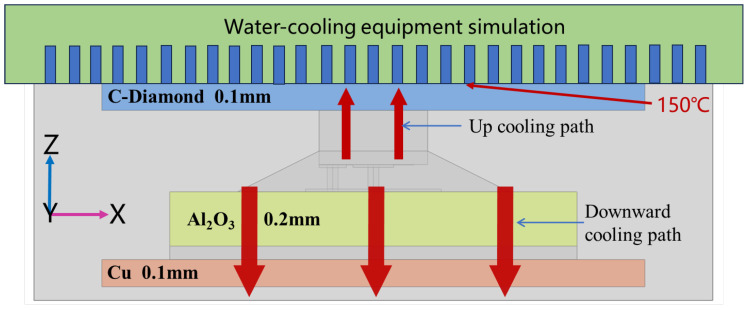
Schematic of the double-sided cooling flip-chip packaging model with water-cooling equipment.

**Figure 8:**
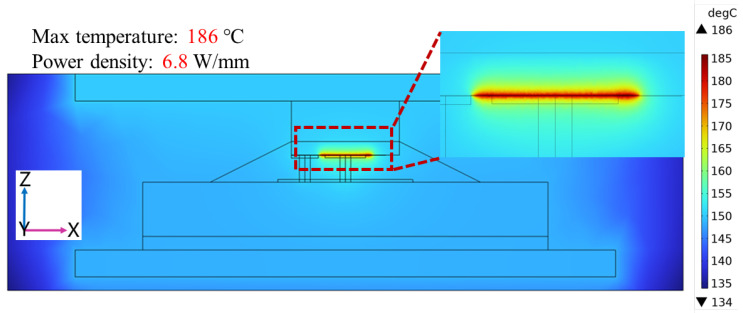
Simulation result of double-sided cooling flip-chip packaging Ga_2_O_3_ chip model with water-cooling equipment at a power density of 6.8 W/mm.

By changing the size of the heat source, the simulation results showed that the maximum power density was 6.8 W/mm at the maximum temperature of 200 °C for the enhanced double-sided cooling flip-chip Ga_2_O_3_ chip (Figure 6). To further improve the heat dissipation of the chip, additional cooling equipment is often added on top of the package, such as air-cooling or water-cooling equipment. This paper assumed that water-cooling equipment on the top of the double-sided cooling flip-chip Ga_2_O_3_ chip could keep the top of the package at 150 °C, and the situation of heat dissipation with water-cooling equipment was simulated by adding boundary temperature conditions (Figure 7). The simulation result is shown in Figure 8. The maximum temperature of the chip was 186 °C with a power density of 6.8 W/mm, which was effectively reduced by 14 °C. Therefore, the double-sided cooling flip-chip packaging structure combined with external cooling equipment will be a feasible and practical solution to the thermal management challenges of high-power Ga_2_O_3_ devices.

## 4. Conclusions

Different Ga_2_O_3_ device packaging structures were simulated and studied in this paper. It was found that the maximum temperature of the single-sided flip-chip packaging was 5 °C lower than that of the wire-bonding packaging at a power density of 3.2 W/mm. In addition, an advanced double-sided cooling flip-chip packaging structure for Ga_2_O_3_ chips was proposed in this paper. The heat of the die could be dissipated from the die downward through the alumina ceramic and Cu base plate and could also be dissipated from the die upward through the diamond material. The simulation results showed that the maximum temperature of the chip with a double-sided cooling flip-chip structure was 7 °C lower than that of the single-sided cooling flip-chip structure and 12 °C lower than that of the traditional wire bonding packaging, which effectively reduced the temperature of the chip. In addition, the maximum power density of 6.8 W/mm could be achieved at the limit of 200 °C. When the top of the package was equipped with water-cooling equipment, the maximum chip temperature was 186 °C at the power density of 6.8 W/mm, which was reduced by 14 °C. It can be seen that the double-sided cooling flip-chip packaging structure combined with external cooling equipment can effectively reduce the heat dissipation of Ga_2_O_3_ chips. As a result, the double-sided cooling flip-chip packaging structure based on diamond material is a very feasible package solution for Ga_2_O_3_ devices and has good application prospects.

## Figures and Tables

**Table 1:** Size and Material Parameters of Wire Bonding and Flip-chip Packaging.

Type	Structure	Size (mm^3^)	ρ (kg/m^3^)	λ/(W/m·K)	C (J/kg·K)
Wire bonding packaging	Si sub	0.4 × 0.4 × 0.15	2329	131	700
	Al_2_O_3_	1.5 × 1.5 × 0.2	3965	35	730
	60Sn-40Pb solder	1.5 × 1.5 × 0.05	9000	50	150
	Cu	2 × 2 × 0.1	8960	400	385
	Plastic mold	2.5 × 2.5 × 0.8	2700	0.2	900
	Copper wire	d = 20 μm	8960	400	385
Flip-chip packaging	Si sub	0.4 × 0.4 × 0.15	2329	131	700
	Al_2_O_3_	1.5 × 1.5 × 0.2	3965	35	730
	60Sn-40Pb solder	1.5 × 1.5 × 0.05	9000	50	150
	Cu	2 × 2 × 0.1	8960	400	385
	Plastic mold	2.5 × 2.5 × 0.8	2700	0.2	900
	Underfill epoxy	0.08	2700	5	900
	Copper pillar	r = 20 μm, h = 99 μm	8960	400	385

**Table 2:** Maximum Temperature Simulation Results of Different Packaging Models.

Packaging Model	Maximum Temperature/°C
Conventional wire bonding	115
Conventional single-sided cooling FC	110
The enhanced double-sided cooling FC	103

## Data Availability

Data is contained within the article.
